# Associations of COVID-19 Hospitalizations, ICU Admissions, and Mortality with Black and White Race and Their Mediation by Air Pollution and Other Risk Factors in the Louisiana Industrial Corridor, March 2020–August 2021

**DOI:** 10.3390/ijerph20054611

**Published:** 2023-03-05

**Authors:** Qingzhao Yu, Wentao Cao, Diana Hamer, Norman Urbanek, Susanne Straif-Bourgeois, Stephania A. Cormier, Tekeda Ferguson, Jennifer Richmond-Bryant

**Affiliations:** 1 Biostatistics Program, Louisiana State University Health Sciences Center, New Orleans, LA 70112, USA; 2 Division of Academic Affairs, Our Lady of the Lake Regional Medical Center, Baton Rouge, LA 70808, USA; 3 Department of Forestry and Environmental Resources, North Carolina State University, Raleigh, NC 27695, USA; 4 Epidemiology Program, Louisiana State University Health Sciences Center, New Orleans, LA 70112, USA; 5 Department of Biological Sciences, Louisiana State University, Baton Rouge, LA 70803, USA; 6 Pennington Biomedical Research Center, Baton Rouge, LA 70808, USA; 7 Center for Geospatial Analytics, North Carolina State University, Raleigh, NC 27695, USA

**Keywords:** COVID-19, SARS-CoV-2, coronavirus, hazardous air pollutants, HAPs, racial disparities

## Abstract

Louisiana ranks among the bottom five states for air pollution and mortality. Our objective was to investigate associations between race and Coronavirus Disease 2019 (COVID-19) hospitalizations, intensive care unit (ICU) admissions, and mortality over time and determine which air pollutants and other characteristics may mediate COVID-19-associated outcomes. In our cross-sectional study, we analyzed hospitalizations, ICU admissions, and mortality among positive SARS-CoV-2 cases within a healthcare system around the Louisiana Industrial Corridor over four waves of the pandemic from 1 March 2020 to 31 August 2021. Associations between race and each outcome were tested, and multiple mediation analysis was performed to test if other demographic, socioeconomic, or air pollution variables mediate the race–outcome relationships after adjusting for all available confounders. Race was associated with each outcome over the study duration and during most waves. Early in the pandemic, hospitalization, ICU admission, and mortality rates were greater among Black patients, but as the pandemic progressed, these rates became greater in White patients. However, Black patients were disproportionately represented in these measures. Our findings imply that air pollution might contribute to the disproportionate share of COVID-19 hospitalizations and mortality among Black residents in Louisiana.

## 1. Introduction

Coronavirus Disease 2019 (COVID-19) severity and mortality have been associated with several vulnerability factors, including comorbidities, environmental exposures, natural disasters, sociodemographic factors, and residence in congregate settings [1,2]. During the first wave of COVID-19 cases in the U.S., transmission in congregate settings was responsible for most disease spread [3], while comorbidities among older residents likely elevated risk of death [4]. The second wave of COVID-19 cases in the U.S. saw disproportionate numbers of severe disease and deaths among Black, Hispanic, Native American, and immigrant population groups [2,5,6]. The third wave may have occurred in part due to asymptomatic transmission in congregate settings including prisons and long-term care facilities, disproportionately impacting Black and Hispanic populations [2].

Soon after the start of the pandemic, some evidence emerged of an association between long-term average air pollution concentrations and the prevalence or severity of COVID-19. Notably, significant associations were observed for long-term average concentration of particulate matter (PM) having a diameter smaller than 2.5 μm (PM_2.5_) with SARS-CoV-2 infection prevalence [7,8,9], COVID-19 disease severity [10], intensive care unit (ICU) admission [11,12], ventilator use [12], and mortality [7,11,12,13]. Associations were also observed for long-term average diesel PM concentration estimates for COVID-19 prevalence and mortality [7]; average nitrogen dioxide (NO_2_) concentrations for prevalence [9,10,14], hospitalization [12], ICU admission [12], ventilator use [12], and mortality [12,14]; ozone (O_3_) concentration for mortality [12]; and hazardous air pollutant indices for respiratory and immunological hazard and mortality [15]. Chen et al. [12] also calculated associations with hospitalization, ICU admission, ventilator use, and mortality for 1-month average concentrations of PM_2.5_ and NO_2_. However, evidence was mixed, with some studies showing no association for NO_2_ [11], O_3_ [7,9,10,14], or PM_2.5_ [14,15]. Although many studies suggested a relationship between air pollutant concentration and COVID-19 outcomes, these studies primarily occurred early in the pandemic. Less is known about the association between air pollutant exposure and COVID-19 over time.

Strategies to respond effectively to public health emergencies such as the COVID-19 pandemic require understanding potential causal pathways for disease outcomes [16,17]. Mediation models can be useful to test how conditions present in populations may influence disease status either directly or indirectly. Disparities in COVID-19 outcomes by race combined with evidence about the relationship between COVID-19 and comorbidities, insurance status, and pollution exposure led to the hypothesis that there is a causal pathway between race and COVID-19 mediated by comorbidities, insurance status, and pollution exposure (Supplemental Figure S1). 

Louisiana parishes routinely score well below the national average for quality of life, morbidity, and mortality indices such as low birthweight, child poverty, and median household income [18]. Based on the most recently available data, Louisiana ranks 46th among the states in air quality given by average daily PM_2.5_, 47th in percent smokers among adults, and 45th in the COVID-19 death rate. For the period of 1 March 2020–31 August 2021, 37.7% of Louisiana’s COVID-19 deaths occurred in people identifying as non-Hispanic Black (hereafter referred to as “Black patients”) [19]. In 2020, 41.7% of Louisiana’s COVID-19 deaths occurred among Black patients, compared with 31.2% of Louisiana residents identifying as Black [20]. This is consistent with a recent analysis that connected disparities, systemic racism, economic stress, and COVID-19 mortality [21].

Given the disproportionate impact of COVID-19 on communities of color in Louisiana and the U.S., the goals of this research were to investigate the association of race and COVID-19 outcomes over time and to identify if exposures to air pollution and other characteristics, if any, may mediate associations of race with COVID-19 hospitalizations, ICU admissions, and mortality. We combined datasets from a Louisiana hospital system distributed across the Industrial Corridor and an air pollution database to include both individual and environmental level risk factors. We investigated factors including race, insurance status, comorbidity, and pollutant exposure for four waves of COVID-19 between 1 March 2020 and 31 August 2021.

## 2. Materials and Methods

### 2.1. Study Population and Health Data

In our cross-sectional study, we evaluated associations between race and COVID-19 hospitalizations, ICU admissions, and mortality and tested for factors that may mediate relationships. We used the Franciscan Missionaries of Our Lady (FMOL) Health System COVID-19 registry to identify patients at ten Louisiana locations distributed across the Industrial Corridor (Supplemental Table S1). The study was approved by the Louisiana State University Health Sciences Center-New Orleans Institutional Review Board (protocol #1986). 

A total of 13,454 patients aged eighteen years or older who tested positive by a polymerase chain reaction (PCR) test for SARS-CoV-2 were identified using the Epic healthcare software between 1 March 2020 and 31 August 2021. This period is broken down by waves: 1 March–10 June 2020 (First Wave), 11 June–6 October 2020 (Second Wave), 7 October 2020–30 June 2021 (Third Wave), and 1 July–31 August 2021 (Fourth Wave). These were chosen to minimize both cases and mortality at the beginning and end of each period using the Johns Hopkins database for Louisiana [22]. 

Patient-level variables included hospital department, SARS-CoV-2 test date, SARS-CoV-2 test result, age, insurance status (private insurance, Medicaid, Medicare, and self-pay), self-reported race, self-reported ethnicity, sex, admission date, discharge date, length of hospital stay, admission status, ICU stay, ICU admission date, ICU discharge date, length of ICU stay, discharge dispatch, body mass index (BMI), presence of comorbidities, census tract, and census block group. Specific comorbidities were not listed consistently in the database, so they were simply recoded as presence (1) or absence (0) of any comorbidities for each patient in the database. To minimize bias in the patient database, negative PCR tests were not included in the database because tests were often obtained for non-medical reasons (e.g., work, travel, recreation, routine medical procedures). 

Records were complete for hospitalization and ICU admission; records were missing for mortality for 171 Black patients and 128 White patients. Data with missing hospitalization, ICU, or mortality information were removed from the dataset. The final sample size was 11,331. Ethnicity data were missing for 9977 patients. A total of 113 patients (<1%) responded that their ethnicity was “Hispanic or Latino/a”, “Mexican, Mexican American, or Chicano”, or “Other Hispanic, Latino/a, or Spanish origin”, while 1271 patients responded that they were “Not of Hispanic or Latino/a or Spanish Origin”. Therefore, ethnicity was not included in the statistical analyses.

### 2.2. Air Pollution Data

Air pollution burden calculations were based on Mikati et al. [23]. Absolute burden for each respiratory hazardous air pollutant was calculated by census tract as the weighted average of the emissions over the block groups within each tract. Facility-level air pollutant emissions data across the state of Louisiana were obtained from the 2017 National Emissions Inventory [24], and data for the census block groups and census tracts, including shape files and demographic characteristics, were obtained from the 2015–2019 American Community Survey [25]. Air pollutant emissions for each facility were assigned to a census block group when the block group’s centroid fell within a 2.5-mile radius of the facility. Air pollution burden was calculated as the sum of assigned facility-level emissions for each block group. Air pollution burden was then summed for each census tract. Air pollutants included PM_2.5_ and hazardous air pollutants (HAPs) known to have respiratory health effects: 1,3-dichloropropene, 2,4-toluene-diisocyanate, acetaldehyde, acrolein, acrylic acid, arsenic, beryllium, cadmium, chlorine, chloroprene, chromium, diesel PM, formaldehyde, hexamethylene-1,6-diisocyanate, hydrazine, hydrochloric acid, naphthalene, nickel, polycyclic organic matter (POM), propylene, and triethylamine. Oil and gas wells and refineries, which are prevalent naphthalene sources, and a neoprene plant, a chloroprene source, fall within the hospital service area (Supplemental Figure S2). Emissions burdens were assigned to 12,031 individual COVID-19 patients in the FMOL Health System database based on their census tract of residence. Bias minimization related to spatial assignment of emissions burdens is described in Mikati et al. [23].

### 2.3. Statistical Analysis

Differences in population characteristics, including air pollutant burden, were first illustrated using summary statistics. Direct relationships of race with other demographic variables (age, sex, BMI, presence of comorbidities, insurance status) or with disease-related variables (hospitalization, ICU admission, mortality) were screened via χ^2^ or ANOVA for categorical or continuous variables, respectively. Patient status was determined using hospital data for admission status, length of hospital stay, ICU status, and length of ICU stay. *p*-value < 0.05 for the χ^2^ or ANOVA test signified a potential significant difference between Black and White COVID-19 patients. 

We used mediation analysis to test for environmental risk factors, called third variables, that might explain widely reported racial disparities in the COVID-19 outcomes. Mediation analysis is used here because it tests for causal associations from the explanatory variable (race) to third variables (environmental risk factors) and then to the outcome (COVID-19 hospitalization, ICU admission, or mortality) to determine if the pollutants are responsible for the association [26,27,28]. Potential mediators that intervene in the associations of race with COVID-19 outcomes (hospitalization, ICU admissions, mortality) were first evaluated. The variables included age, insurance status (private insurance, Medicaid, Medicare, and self-pay), ethnicity, sex, presence of comorbidities, and pollutant emissions. ANOVA or χ^2^ testing was performed to check the relationship between race and each variable, and between each variable and health outcomes. Potential mediators and potential covariates in the association between race and health effect were identified. Associations of each variable with both race and health effect indicated that the variable is a potential mediator. Variables associated with just health effects but not with race were identified as covariates to be controlled in the mediation analysis. Mediation analysis was then used to test if a portion of the race–outcome relationship could be accounted for by each intermediate variable after adjusting for all potential mediators, covariates, and confounders [26,27,28]. Significant mediators with the same sign as the total effect were considered as part of the racial differences explained by the mediator, while those with opposite sign suggested that the potential mediator caused greater uncertainty. 

We used the R software v4.0.5 for data organization (packages *dplyr*, *tidyr*, *bit65*, and *data.table*) and for the merger of geographic data with air pollution emissions data and output of shape files containing emissions burdens (packages *tigris*, *Hmisc*, *sp*, and *rgdal*). The R package *mma* was used to perform the mediation analysis [29]. Confidence balls [30] were created to control the overall confidence level at 95%. We confirmed each of the criteria listed under the STrengthening the Reporting of OBservational Studies in Epidemiology checklist for cross-sectional studies during completion of this manuscript [31].

## 3. Results

Of the 11,331 patients in the final sample, 5708 (50.4%) identified as non-Hispanic Black, and 5623 (49.6%) identified as non-Hispanic White (Table 1). In comparison, 33.8% of the population of Louisiana census tracts associated with patients’ residential addresses (referred to hereafter as the “patient population”) identified as non-Hispanic Black, and 58.8% identified as non-Hispanic White. Census tract population data were available for 89% of patients. A total of 6210 (54.8%) cases identified as female, and 5119 (45.2%) identified as male. On average, Black patients were 7.9 years younger than White patients. Black patients had a higher average BMI (*p*-value < 2 × 10^−16^), but average BMI for both groups was in the obese range (BMI > 30). Length of hospital and ICU stays were both significantly higher among White patients, although that difference diminished for Medicare recipients and those without insurance. More Black patients had Medicaid (61.9%) or were uninsured (61.6%), while more White patients had private insurance (62.5%) or Medicare (59.4%). Among the twenty-two pollutants tested, emissions burden was statistically significantly higher for Black patients in seventeen compounds and for White patients in three compounds, with no significant difference for two pollutants, hydrazine and propylene.

For the study duration, hospitalizations were significantly higher among White patients (53.4%), while ICU admissions were significantly higher among Black patients (52.4%). Table 2 provides the frequency of hospital and ICU admissions and deaths for the full study period and for each wave of the study. Equitable Black and equitable White indicate the ratio of the share of the population of patients in each group compared with the number of patients that would be expected for each group based on the proportion of each group in the Louisiana census tracts sending patients to the FMOL Health System. Compared with their share of the patient population, Black patients were over-represented among hospitalizations by 28%, among ICU admissions by 43%, and among total COVID-19 patients by 38% (Table 2). Hospital and ICU admissions significantly exceeded the share of the population for Black patients by 86% and 89%, respectively, during the first wave and by 40% and 56%, respectively, during the second wave. By the third wave, the proportions of hospital and ICU admissions were higher among White patients with a significant χ^2^, but the proportion of hospital and ICU admissions among Black patients were 16% and 36% greater, respectively, than the share of the population identifying as Black.

Information regarding mortality (patients who expired while at the hospital or within 7 days of discharge) was available for 11,032 (97.3%) cases (Table 2). For the study duration, the proportion of those who died was significantly higher for White patients, but the proportion of Black patients who died was still 25% greater than the proportion of Black people in the Louisiana census tracts sending patients to the FMOL Health System. The proportion of patients who died was nearly 65% for Black patients during the first wave, with the share of the patient population that is Black over-represented by 78%, but was significantly higher for White patients during the second and third waves and not significantly different in the fourth wave. During the second wave, mortality among Black patients was still 28% higher than the share of patient population identifying as Black.

**Figure 1:**
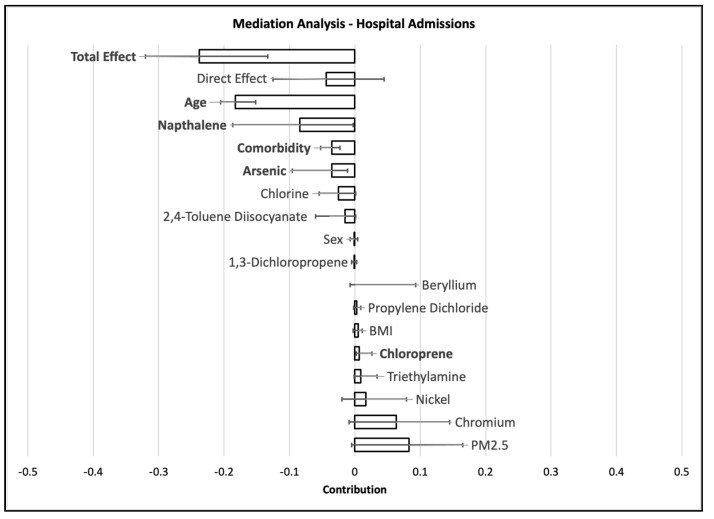
Mediation analysis results for hospitalizations. Whiskers indicate the 95% confidence interval around the mediation effect, with each tested mediator shown by a column. Statistically significant effects are bolded. A positive effect suggests a larger effect in Black patients compared with White patients, and a negative effect suggests a larger effect in White patients compared with Black patients.

**Figure 2:**
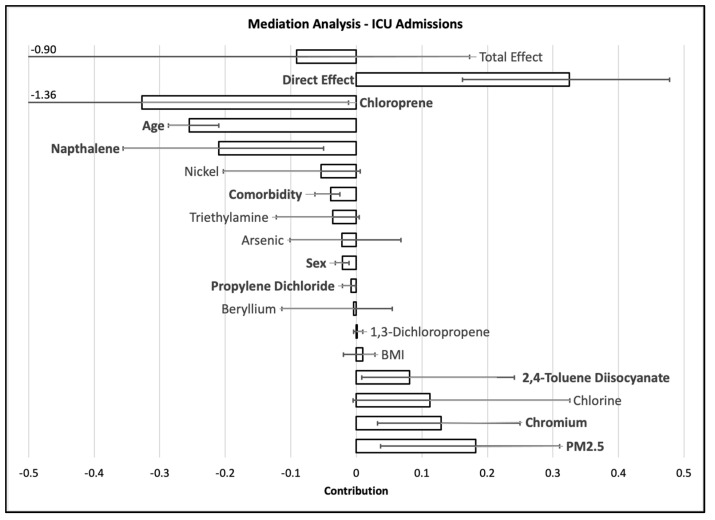
Mediation analysis results for ICU admissions. Whiskers indicate the 95% confidence interval around the mediation effect, with each tested mediator shown by a column. Where the lower confidence interval goes beyond the data range shown on the page, the lower bound is provided numerically on the graph. Statistically significant effects are bolded. A positive effect suggests a larger effect in Black patients compared with White patients, and a negative effect suggests a larger effect in White patients compared with Black patients.

**Figure 3:**
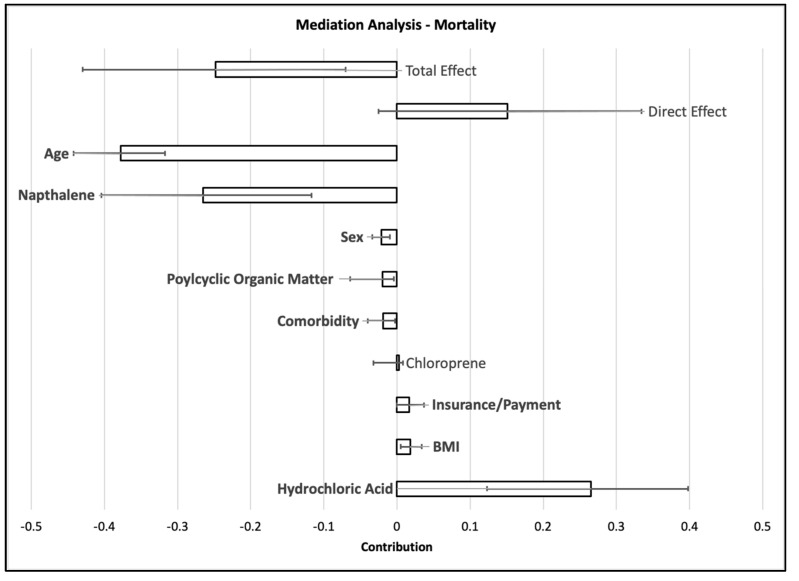
Mediation analysis results for mortality. Whiskers indicate the 95% confidence interval around the mediation effect, with each tested mediator shown by a column. Statistically significant effects are bolded. A positive effect suggests a larger effect in Black patients compared with White patients, and a negative effect suggests a larger effect in White patients compared with Black patients.

The mediation analysis figures (Figure 1, Figure 2 and Figure 3 and Figures S3–S14) illustrate the relative relationships between effect estimates for Black and White patients and how much the health effect (hospital admissions, ICU admissions, or mortality) can be explained by other factors. Based on the coding (1 = White, 2 = Black), a positive total effect suggests a larger effect in Black patients compared with White patients, and a negative total effect suggests a larger effect in White patients compared with Black patients. The direct effect illustrates how much of the health effect with respect to race can be explained only by race. The other effects show how much the health effect with respect to race can be explained by other factors, such as age, sex, comorbidity, or air pollution. For each factor, an effect that is the same sign as the total effect with a confidence interval that does not include zero suggests that the specific factor can explain some of the race–health effect relationship. An effect with a sign that is different from the total effect and/or large confidence intervals can suggest large uncertainty in the total effect or may indicate that a direct effect or mediated effect may partially explain effect on a different race than is represented in the total effect.

Age and, with a smaller contribution, presence of comorbidities were significant mediators of the race–hospitalization relationship (Figure 1) for the entire study period. The negative sign of the total effect and direct effect indicated greater hospital admissions among White patients, with age and comorbidities as significant mediators for each wave. Naphthalene and arsenic were significant mediators of the total effect for the duration of the study. Naphthalene was not a significant mediator for any of the individual waves, and arsenic was only for the fourth wave. PM_2.5_ and chromium exposures may have increased the effect among Black patients. However, these exposures may have added uncertainty to the race–hospitalizations total effect because the different sign of these mediation coefficients widened the confidence intervals around the total effect. 

The model for race–ICU admission for the entire study period (Figure 2) included a direct effect that was larger than and opposite in sign to total effect, widening the confidence interval around total effect to suggest uncertainty. The direct effect of different sign may suggest that mediating factors, such as age, comorbidity, sex, and exposure to chloroprene, naphthalene, and propylene dichloride, may contribute to a greater total effect in White patients but that Black patients may be more likely to experience COVID-19 ICU admissions in the absence of the mediating factors. PM_2.5_ and chromium emissions burden potentially contribute to a greater effect in Black patients but widened the confidence intervals around total effect. Age was a mediator of the race–ICU admission effect during each wave. During the third wave, the total effect between race and ICU admission was near zero, but there was a greater direct effect on Black patients and greater indirect effect of PM_2.5_ emissions on Black patients balanced by greater indirect effects of age, cadmium emissions, and nickel emissions on White patients. The fourth wave produced a large total effect for the race–ICU admission model that included a direct effect comprising more than half of the total effect and indirect effects from age, insurance status, sex, and emissions of POM.

The mediation analysis results indicate that for the total duration and for each wave, there was a greater total effect in White patients, with age consistently a significant mediator of the total effect of race on mortality (Figure 3). The direct effect of different sign may suggest that being of Black race predicts a greater race-based mortality effect in COVID-19 patients, and the greater total mortality effect in White patients may have been driven by mediating factors. Sex and comorbidities had smaller indirect effects for the entire study period but were still significant. Naphthalene was identified as a mediator of the total effect, contributing to a greater effect in White patients for the total duration, while hydrochloric acid added uncertainty to the assessment of mediation. Hydrochloric acid burden may have contributed to the effect in Black patients. Naphthalene was identified as a potential mediator during the first wave but was not significant and added uncertainty to that model. POM was a significant mediator of the race–mortality relationship during the fourth wave. POM emerged as a potential mediator in the total duration model but was of small magnitude.

## 4. Discussion

A complicated picture of racial disparities in COVID-19 hospitalization, ICU admission, and death emerges from these results. For the entire study period, hospitalization and mortality rates among those who were diagnosed with COVID-19 in Louisiana’s Industrial Corridor were greater for White patients than for Black patients, while ICU admission rates were higher for Black patients. These proportions shifted towards White patients by late 2020. However, the proportion of those diagnosed with COVID-19 as well as those hospitalized, admitted to the ICU, and who died remained disproportionately higher for Black patients compared with the patients’ residential areas, despite the 7.9-year age difference between Black and White patients. For example, across the entire study period, COVID-19 mortality among Black patients was 25% greater than what would be anticipated based on the proportion of the patient population identifying as Black, while COVID-19 mortality among White patients was 14% below what would be anticipated based on the patient population identifying as White. 

Among the population of those who had to be hospitalized due to COVID-19, most of the association of race could be explained by mediators, i.e., third variables. Age was the strongest mediator, accounting for the largest share of the association between race and COVID-19 hospitalization. In each wave, the average age of Black patients was 8–9 years younger than the average age of White patients. In fact, life expectancy for Black Louisiana residents is 3.4 years shorter than for White Louisiana residents [32]. These factors make it difficult to disentangle the effect of race from the effect of age. Cronin and Evans [33] calculated the U.S. COVID-19 mortality rate throughout 2020 by race-ethnicity and age and found higher mortality for Black males and females for every age group (0–44 y, 45–64 y, 65–74 y, and 75+ y) with a greater effect of age than race or sex.

Findings that naphthalene and chloroprene explained part of the associations between White race and ICU admissions and that naphthalene also explained part of the associations of White race with hospital admissions and mortality were surprising given that their burdens among Black patients in Louisiana were 8.9 and 4.5 times higher, respectively, than for White patients. Chlorine was found to explain ICU admissions among Black patients, and hydrochloric acid was found to explain mortality among Black patients. These findings are consistent with chlorine’s burden being 17 times greater and hydrochloric acid’s burden being 8.0 times greater among Black patients than White patients. Terrell and James [15] noted higher COVID-19 incidence in locations with a higher respiratory hazard index, where the index was computed by the U.S. EPA based on HAPs emissions. PM_2.5_ explained ICU admissions and mortality among Black patients and was 5.2 times greater among Black patients compared with White patients. Several studies [7,11,12,13] found associations of PM_2.5_ with COVID-19 using data from the first few months of the pandemic, but they either used a nationwide domain or studied different parts of the country. Sidell et al. [9] studied how the relationship between air pollution and COVID-19 infection changed in a southern California cohort over four waves spanning 1 March 2020 through 28 February 2021. They observed associations to persist for each wave and the entire duration of their study for both 1-month average and 1-year average PM_2.5_ and NO_2_ concentrations and between COVID-19 infection and 1-year average O_3_ concentrations for the second, third, and fourth waves and entire study duration. However, the magnitude of the associations declined over the third and fourth waves, especially for PM_2.5_. Uncertainties persist about the influence of air pollution on COVID-19 outcomes over the course of the pandemic. Terrell and James [15] calculated a correlation of 0.21 for PM_2.5_ concentration with COVID-19 mortality for Louisiana, and Xu et al. [34] noted for a study of COVID-19 in Texas that PM_2.5_ concentrations were not associated with COVID-19 mortality.

There were some limitations specific to this dataset. These analyses reflect the data and results of the full population that interfaced with the FMOL Health System based primarily in the Industrial Corridor. This selective population was not representative of all Louisiana COVID-19 hospitalizations and thus limits some generalizability of our results for the full state. The most recent HAP emission data were from 2017. Additionally, vaccination status was not included in the dataset but could have affected severe outcomes during the last two waves.

Mediation analysis showed a clear relationship between race and outcome at the beginning of the pandemic, but race appeared less influential over time. Mediation analyses highlighted the uncertainty in the race–outcome relationships across waves. Although several air pollutants were associated with race, with higher emissions burdens among predominantly Black census tracts, air pollution did not appear to consistently mediate the total race–outcome relationship for most waves. Uncertainties in the mediation analyses raise questions about unmeasured confounding. VanderWeele [35] asserted four necessary assumptions for mediation analysis: (1) control for confounding of the exposure–outcome relationship, (2) control for confounding of the mediator–outcome relationship, (3) control for confounding of the exposure–mediator relationship, and (4) no confounder of the mediator–outcome relationship is affected by the exposure. The first three were accomplished through the process of checking for significant associations among the exposure, potential mediator, and outcome. However, the final assumption is more difficult to enforce for this study given that long-standing racialization may introduce other, uncontrolled factors [36]. Similarly, it is difficult to ascertain whether any mediators were omitted from the analysis. Additionally, exposure measurement error or exposure misclassification has the potential to weaken the associations between the exposure and mediators. In the case of the HAP burdens, Mikati et al. [23] sought to control this by testing different assignment radii and found little difference. Use of census tract-level assignments also helps to localize the exposure estimates.

## 5. Conclusions

The wave-by-wave results of this study indicate that the role of race in the associations of COVID-19 outcomes has evolved over the course of the pandemic in Louisiana. Early in the pandemic, the association of race with hospitalization, ICU admission, and mortality appeared to be mediated by age. However, the younger age profile of Black COVID-19 patients contradicts findings of enhanced risk to older patients [33], suggesting that race rather than age played a role, especially early in the pandemic. As time went on, the analysis revealed greater impact on White patients in terms of overall numbers, but still with a disproportionate impact on Black patients compared with the local population. These findings reveal a need for strategies that focus on disadvantaged communities and individuals to protect each population group from exposure to the SARS-CoV-2 virus and from the severe impacts of COVID-19. Our findings also highlight a need to disentangle the associations of COVID-19 outcomes with race as a marker for measures of disadvantage and social determinants of health.

Burden from air pollutants may have explained some of the race–outcome associations. Findings that greater effect of chlorine and PM_2.5_ in Black patients on ICU admissions and greater effect of hydrochloric acid in Black patients on mortality were not surprising because their burdens among Black patients were 17, 5.2, and 8.0 times higher, respectively, than among White patients. Our results suggest that disparities in environmental conditions may have exacerbated inequities in COVID-19 impacts among Black patients.

## Figures and Tables

**Table 1:** Characteristics of the Louisiana Franciscan Missionaries of Our Lady (FMOL) Health System COVID-19 patient hospitalizations during March 2020–August 2021 and air pollutant exposures.

COVID-19 Hospitalizations	Black (*N* = 5708) Mean ± SD	White (*N* = 5623) Mean ± SD	*p* ^1^
**Age (y)**	48.5 ± 18.9	**56.4** ± 20.0	<2 × 10^−16^
**Hospital length of stay (d)**	5.70 ± 8.80	**6.82** ± 9.75	2.40 × 10^−6^
**ICU length of stay (d)**	5.75 ± 6.51	**7.96** ± 9.80	7.13 × 10^−7^
BMI	31.7 ± 9.89	30.6 ± 9.16	0.128
Private Insurance (*n*)	924	1538	<2 × 10^−16^
Medicaid (*n*)	1948	1198	
Medicare (*n*)	1329	1947	
Self-Pay (*n*)	1507	940	
Hazardous Air Pollutants^+^			
**Dichloro:1,3-dichloropropene**	0.104	**0.233**	1.28 × 10^−10^
**2,4-toluene**	**0.0324**	0.0143	9.10 × 10^−8^
**Acetaldehyde**	4120	**4640**	1.29 × 10^−13^
**Acrolein**	**495**	272	<2 × 10^−16^
**Acrylic Acid**	**50.8**	19.1	1.62 × 10^−8^
**Arsenic**	**2.33**	1.00	<2 × 10^−16^
**Beryllium**	**0.338**	0.109	<2 × 10^−16^
**Cadmium**	**3.63**	0.842	<2 × 10^−16^
**Chlorine**	**3680**	216	<2 × 10^−16^
**Chloroprene**	**231**	50.9	<2 × 10^−16^
**Chromium**	**2.46**	0.443	<2 × 10^−16^
**Diesel PM**	**0.293**	0.0481	<2 × 10^−16^
**Formaldehyde**	**4430**	1830	<2 × 10^−16^
**HCl**	**21,700**	2720	<2 × 10^−16^
**Hexamethylene 6-diisocyanate**	0.410	**2.89**	<2 × 10^−16^
Hydrazine	0.00231	0.0152	0.565
**Naphthalene**	**2280**	255	<2 × 10^−16^
**Nickel**	**95.3**	23.1	<2 × 10^−16^
**PM2.5**	**83.0**	16.1	<2 × 10^−16^
**Polycyclic organic matter**	**0.0231**	0.00793	1.47 × 10^−5^
Propylene	8.90	6.44	0.289
**Triethylamine**	**46.6**	19.0	<2 × 10^−16^

^1^ *p*-values were calculated using square root transformed data to normalize the data distribution; standard deviation not provided for the Hazardous Air Pollutants because the pollutant data were not normally distributed. **Bold** type indicates statistically significance differences, with the higher values in bold type. ICU = intensive care unit; BMI = body mass index; PM = particulate matter; HCl = hydrogen chloride; SD = standard deviation.

**Table 2:** Count tables for χ^2^ analysis for each wave of the study and for the full study period for Louisiana Franciscan Missionaries of Our Lady (FMOL) Health System COVID-19 patient hospital admissions (HA), intensive care unit admissions (ICU), and death outcomes, by race and population ratio for March 2020–August 2021.

	Hospital Admission	ICU Admission	No Hospital Admission	Death	No Death
*Full Study Period: 1 March 2020–31 August 2021*					
Black	1621	**726**	**3361**	289	**5248**
Equitable Black	***1.28***	***1.43***	***1.42***	***1.25***	***1.38***
White	**1859**	660	3104	**344**	5151
Equitable White	0.84	0.75	0.76	0.86	0.78
*p*-value			5.04 × 10^−7^		0.0209
*First Wave: 1 March 2020–10 June 2020*					
Black	**256**	**192**	**314**	**107**	**655**
Equitable Black	***1.86***	***1.89***	***2.10***	***1.78***	***1.99***
White	121	86	96	58	245
Equitable White	0.51	0.49	0.37	0.55	0.43
*p*-value			0.0149		0.0475
*Second Wave: 11 June 2020–6 October 2020*					
Black	**307**	**188**	**645**	58	**1082**
Equitable Black	***1.40***	***1.56***	***1.64***	***1.28***	***1.57***
White	294	143	433	66	804
Equitable White	0.77	0.68	0.63	0.84	0.67
*p*-value			2.43 × 10^−3^		0.0269
*Third Wave: 7 October 2020–30 June 2021*					
Black	619	287	**1178**	83	1921
Equitable Black	***1.16***	***1.36***	***1.42***	0.98	***1.34***
White	**843**	**290**	1089	**148**	**2015**
Equitable White	0.91	0.79	0.76	**1.01**	0.81
*p*-value			5.47 × 10^−8^		1.85 × 10^−4^
*Fourth Wave: 1 July 2021–31 August 2021*					
Black	439	59	1224	41	1590
Equitable Black	***1.16***	0.81	***1.24***	0.99	***1.18***
White	**601**	**141**	**1486**	72	2087
Equitable White	0.91	***1.11***	0.86	1.00	0.89
*p*-value			5.31 × 10^−5^		0.169

**Bold** type indicates statistical significance. Equitable Black and equitable White indicate the ratio of the share of the population of patients in each group compared with the number of patients that would be expected for each group based on the proportion of each group in the Louisiana census tracts sending patients to the FMOL Health System. *Italic* type indicates an equitable Black or equitable White ratio above one.

## Data Availability

Restrictions apply to the availability of these data. Data were obtained from the FMOL Health System and are available from the authors with the permission of FMOL.

## Supplementary Materials

The following supporting information can be downloaded at: https://www.mdpi.com/article/10.3390/ijerph20054611/s1, Figure S1: Hypothetical causal pathways showing that the association between race and COVID-19 may be mediated by comorbidities, insurance status, and pollution exposure; Figure S2: Locations of a chloroprene point source and of dispersed naphthalene sources in the region of southern Louisiana feeding patients to the Franciscan Missionaries of Our Lady Health System; Figure S3: Mediation analysis results for hospitalizations, 1 March–10 June 2020. Whiskers indicate the 95% confidence interval around the mediation effect, with each tested mediator shown by a column. Statistically significant effects are bolded. Positive total effect suggests a larger effect in Black patients compared with White patients, and a negative total effect suggests a larger effect in White patients compared with Black patients; Figure S4: Mediation analysis results for hospitalizations, 11 June–6 October 2020. Whiskers indicate the 95% confidence interval around the mediation effect, with each tested mediator shown by a column. Statistically significant effects are bolded. Positive total effect suggests a larger effect in Black patients compared with White patients, and a negative total effect suggests a larger effect in White patients compared with Black patients; Figure S5: Mediation analysis results for hospitalizations, 7 October 2020–30 June 2021. Whiskers indicate the 95% confidence interval around the mediation effect, with each tested mediator shown by a column. Statistically significant effects are bolded. Positive total effect suggests a larger effect in Black patients compared with White patients, and a negative total effect suggests a larger effect in White patients compared with Black patients; Figure S6: Mediation analysis results for hospitalizations, 1 July–31 August 2021. Whiskers indicate the 95% confidence interval around the mediation effect, with each tested mediator shown by a column. Statistically significant effects are bolded. Positive total effect suggests a larger effect in Black patients compared with White patients, and a negative total effect suggests a larger effect in White patients compared with Black patients; Figure S7: Mediation analysis results for ICU admissions, 1 March–10 June 2020. Whiskers indicate the 95% confidence interval around the mediation effect, with each tested mediator shown by a column. Where the lower confidence interval goes beyond the data range shown on the page, the lower bound is provided numerically on the graph. Statistically significant effects are bolded. Positive total effect suggests a larger effect in Black patients compared with White patients, and a negative total effect suggests a larger effect in White patients compared with Black patients; Figure S8: Mediation analysis results for ICU admissions, 11 June–6 October 2020. Whiskers indicate the 95% confidence interval around the mediation effect, with each tested mediator shown by a column. Where the lower confidence interval goes beyond the data range shown on the page, the lower bound is provided numerically on the graph. Statistically significant effects are bolded. Positive total effect suggests a larger effect in Black patients compared with White patients, and a negative total effect suggests a larger effect in White patients compared with Black patients; Figure S9: Mediation analysis results for ICU admissions, 7 October 2020–30 June 2021. Whiskers indicate the 95% confidence interval around the mediation effect, with each tested mediator shown by a column. Where the lower confidence interval goes beyond the data range shown on the page, the lower bound is provided numerically on the graph. Statistically significant effects are bolded. Positive total effect suggests a larger effect in Black patients compared with White patients, and a negative total effect suggests a larger effect in White patients compared with Black patients; Figure S10: Mediation analysis results for ICU admissions, 1 July–31 August 2021. Whiskers indicate the 95% confidence interval around the mediation effect, with each tested mediator shown by a column. Where the lower confidence interval goes beyond the data range shown on the page, the lower bound is provided numerically on the graph. Statistically significant effects are bolded. Positive total effect suggests a larger effect in Black patients compared with White patients, and a negative total effect suggests a larger effect in White patients compared with Black patients; Figure S11: Mediation analysis results for mortality, 1 March–10 June 2020. Whiskers indicate the 95% confidence interval around the mediation effect, with each tested mediator shown by a column. Statistically significant effects are bolded. Positive total effect suggests a larger effect in Black patients compared with White patients, and a negative total effect suggests a larger effect in White patients compared with Black patients; Figure S12: Mediation analysis results for mortality, 11 June–6 October 2020. Whiskers indicate the 95% confidence interval around the mediation effect, with each tested mediator shown by a column. Statistically significant effects are bolded. Positive total effect suggests a larger effect in Black patients compared with White patients, and a negative total effect suggests a larger effect in White patients compared with Black patients; Figure S13: Mediation analysis results for mortality, 7 October 2020–30 June 2021. Whiskers indicate the 95% confidence interval around the mediation effect, with each tested mediator shown by a column. Statistically significant effects are bolded. Positive total effect suggests a larger effect in Black patients compared with White patients, and a negative total effect suggests a larger effect in White patients compared with Black patients; Figure S14: Mediation analysis results for mortality, 1 July–31 August 2021. Whiskers indicate the 95% confidence interval around the mediation effect, with each tested mediator shown by a column. Statistically significant effects are bolded. Positive total effect suggests a larger effect in Black patients compared with White patients, and a negative total effect suggests a larger effect in White patients compared with Black patients. Table S1: List of hospitals in the Franciscan Missionaries of Our Lady Health System.
