# Supplementary material for: Associations of COVID-19 Hospitalizations, ICU Admissions, and Mortality with Black and White Race and Their Mediation by Air Pollution and Other Risk Factors in the Louisiana Industrial Corridor, March 2020–August 2021
Source: Int J Environ Res Public Health. 2023 Mar 5;20(5):4611. doi: 10.3390/ijerph20054611 (PMC10001987; doi:10.3390/ijerph20054611)
Supplement: Supplementary file 1 [file ijerph-20-04611-s001.zip › ijerph-2250581-Supplementary Materials.pdf]

Supplemental Material

Risk factors among Black and White COVID-19 patients from a Louisiana Hospital System, March, 2020 – August, 2021

Contents

TABLES.. 4

Table S1: List of hospitals in the Franciscan Missionaries of Our Lady Health System... 4

FIGURES.. 5

Figure S1: Hypothetical causal pathways showing that the association between race and COVID-19 may be mediated by comorbidities, insurance status, and pollution exposure.. 5

Figure S2: Locations of a chloroprene point source and of disperse naphthalene sources in the region of Southern Louisiana feeding patients to the Franciscan Missionaries of Our Lady Health System.. 6

Figure S3: Mediation analysis results for hospitalizations, March 1 – June 10, 2020. Whiskers indicate the 95% confidence interval around the mediation effect, with each tested mediator shown by a column. Statistically significant effects are bolded. Positive total effect suggests a larger effect in Black patients compared with White patients, and a negative total effect suggests a larger effect in White patients compared with Black patients.................. 7

Figure S4: Mediation analysis results for hospitalizations, June 11 – October 6, 2020. Whiskers indicate the 95% confidence interval around the mediation effect, with each tested mediator shown by a column. Statistically significant effects are bolded. Positive total effect suggests a larger effect in Black patients compared with White patients, and a negative total effect suggests a larger effect in White patients compared with Black patients.................. 8

Figure S5: Mediation analysis results for hospitalizations, October 7, 2020 – June 30, 2021. Whiskers indicate the 95% confidence interval around the mediation effect, with each tested mediator shown by a column. Statistically significant effects are bolded. Positive total effect suggests a larger effect in Black patients compared with White patients, and a negative total effect suggests a larger effect in White patients compared with Black patients.................. 9

Figure S6: Mediation analysis results for hospitalizations, July 1 – August 31, 2021. Whiskers indicate the 95% confidence interval around the mediation effect, with each tested mediator shown by a column. Statistically significant effects are bolded. Positive total effect suggests a larger effect in Black patients compared with White patients, and a negative total effect suggests a larger effect in White patients compared with Black patients.................. 10

Figure S7: Mediation analysis results for ICU admissions, March 1 – June 10, 2020. Whiskers indicate the 95% confidence interval around the mediation effect, with each tested mediator shown by a column. Where the lower confidence interval goes beyond the data range shown on the page, the lower bound is provided numerically on the graph. Statistically significant effects are bolded. Positive total effect suggests a larger effect in Black patients compared with White patients, and a negative total effect suggests a larger effect in White patients compared with Black patients……………………………………….. 11
Figure S8: Mediation analysis results for ICU admissions, June 11 – October 6, 2020. Whiskers indicate the 95% confidence interval around the mediation effect, with each tested mediator shown by a column. Where the lower confidence interval goes beyond the data range shown on the page, the lower bound is provided numerically on the graph. Statistically significant effects are bolded. Positive total effect suggests a larger effect in Black patients compared with White patients, and a negative total effect suggests a larger effect in White patients compared with Black patients……………………………………….. 12
Figure S9: Mediation analysis results for ICU admissions, October 7, 2020 – June 30, 2021. Whiskers indicate the 95% confidence interval around the mediation effect, with each tested mediator shown by a column. Where the lower confidence interval goes beyond the data range shown on the page, the lower bound is provided numerically on the graph. Statistically significant effects are bolded. Positive total effect suggests a larger effect in Black patients compared with White patients, and a negative total effect suggests a larger effect in White patients compared with Black patients……………………………………….. 13
Figure S10: Mediation analysis results for ICU admissions, July 1 – August 31, 2021. Whiskers indicate the 95% confidence interval around the mediation effect, with each tested mediator shown by a column. Where the lower confidence interval goes beyond the data range shown on the page, the lower bound is provided numerically on the graph. Statistically significant effects are bolded. Positive total effect suggests a larger effect in Black patients compared with White patients, and a negative total effect suggests a larger effect in White patients compared with Black patients……………………………………….. 14
Figure S11: Mediation analysis results for mortality, March 1 – June 10, 2020. Whiskers indicate the 95% confidence interval around the mediation effect, with each tested mediator shown by a column. Statistically significant effects are bolded. Positive total effect suggests a larger effect in Black patients compared with White patients, and a negative total effect suggests a larger effect in White patients compared with Black patients…………………….. 15
Figure S12: Mediation analysis results for mortality, June 11 – October 6, 2020. Whiskers indicate the 95% confidence interval around the mediation effect, with each tested mediator shown by a column. Statistically significant effects are bolded. Positive total effect suggests a larger effect in Black patients compared with White patients, and a negative total effect suggests a larger effect in White patients compared with Black patients…………………….. 16
Figure S13: Mediation analysis results for mortality, October 7, 2020 – June 30, 2021. Whiskers indicate the 95% confidence interval around the mediation effect, with each tested mediator shown by a column. Statistically significant effects are bolded. Positive total effect 17

suggests a larger effect in Black patients compared with White patients, and a negative total effect suggests a larger effect in White patients compared with Black patients………………

Figure S14: Mediation analysis results for mortality, July 1 – August 31, 2021. Whiskers indicate the 95% confidence interval around the mediation effect, with each tested mediator shown by a column. Statistically significant effects are bolded. Positive total effect suggests a larger effect in Black patients compared with White patients, and a negative total effect suggests a larger effect in White patients compared with Black patients…………………….. 18

TABLES

Table S1: List of hospitals in the Franciscan Missionaries of Our Lady Health System

**Hospital**	**Location**
Our Lady of Angels Hospital	Bogalusa, LA
Our Lady of the Lake Ascension	Gonzales, LA
Our Lady of the Lake Assumption Community Hospital	Napoleonville, LA
Our Lady of the Lake Children's Hospital	Baton Rouge, LA
Our Lady of the Lake Regional Medical Center	Baton Rouge, LA
Our Lady of the Lake Regional Medical Center - Livingston	Walker, LA
Our Lady of Lourdes Regional Medical Center	Lafayette, LA
Our Lady of Lourdes Women's and Children's Hospital	Lafayette, LA
St. Elizabeth Hospital	Gonzales, LA
St. Francis Medical Center	Monroe, LA

FIGURES

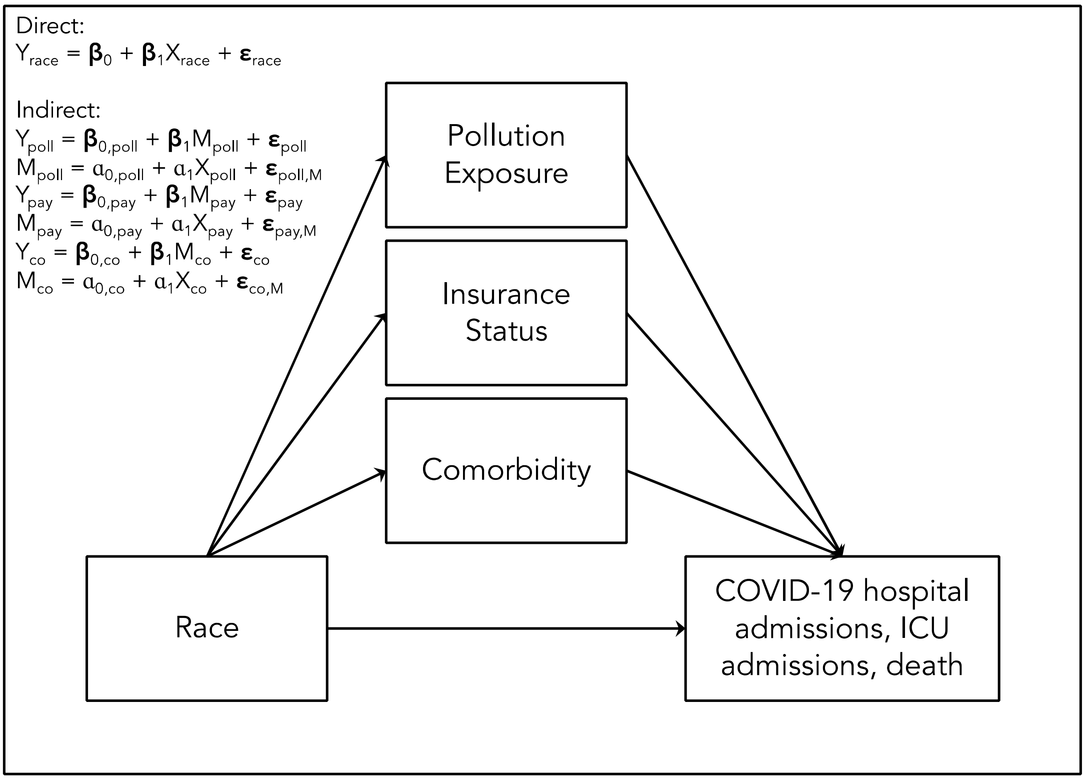

Figure S1: Hypothetical causal pathways showing that the association between race and COVID-19 may be mediated by comorbidities, insurance status, and pollution exposure.

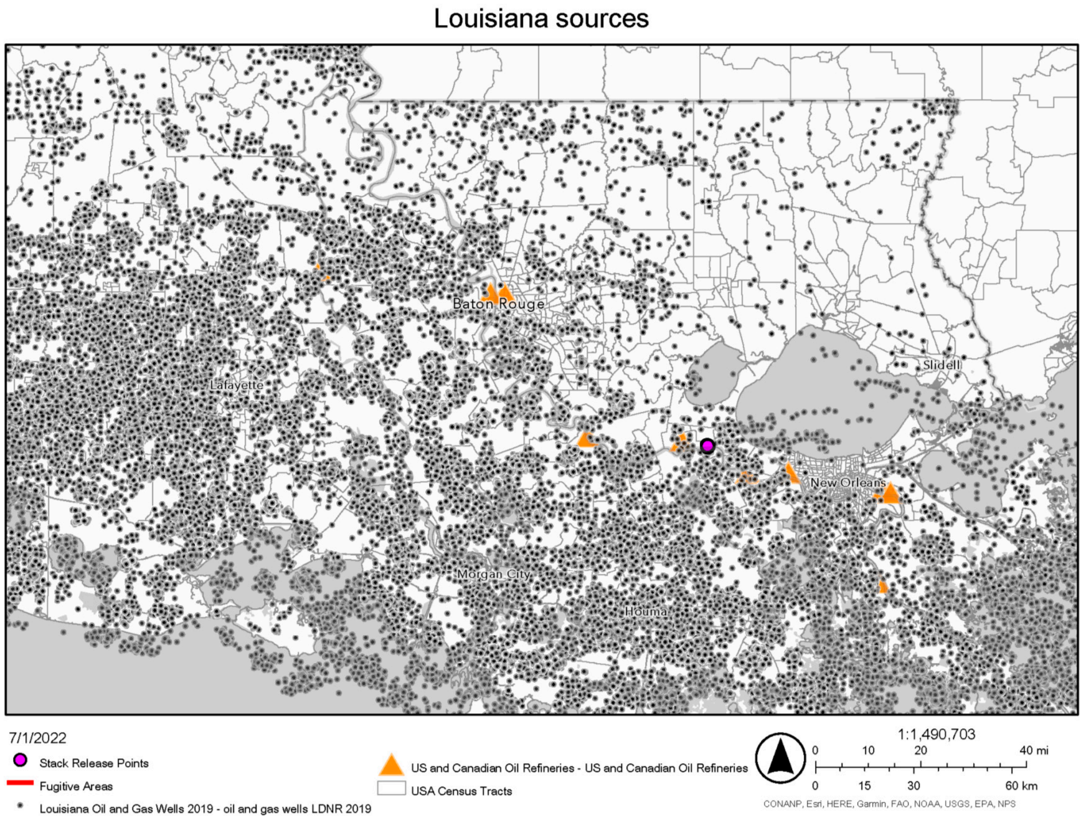

Figure S2: Locations of a chloroprene point source (purple stack release from one major emitting facility) and of disperse naphthalene sources (black dots to show oil and gas wells, orange triangles to show oil refineries) in the region of Southern Louisiana feeding patients to the Franciscan Missionaries of Our Lady Health System.

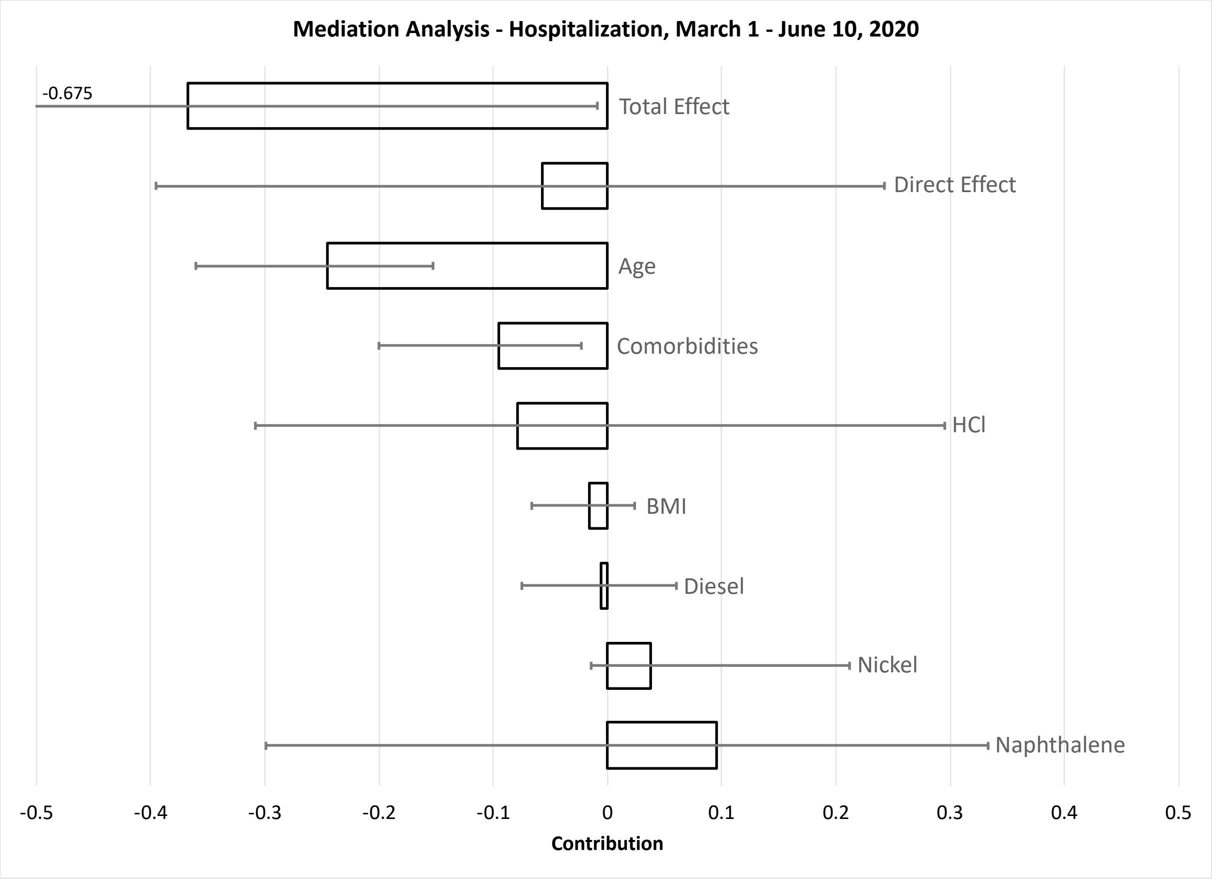

Figure S3: Mediation analysis results for hospitalizations, March 1 – June 10, 2020. Whiskers indicate the 95% confidence interval around the mediation effect, with each tested mediator shown by a column. Statistically significant effects are bolded. Positive total effect suggests a larger effect in Black patients compared with White patients, and a negative total effect suggests a larger effect in White patients compared with Black patients.

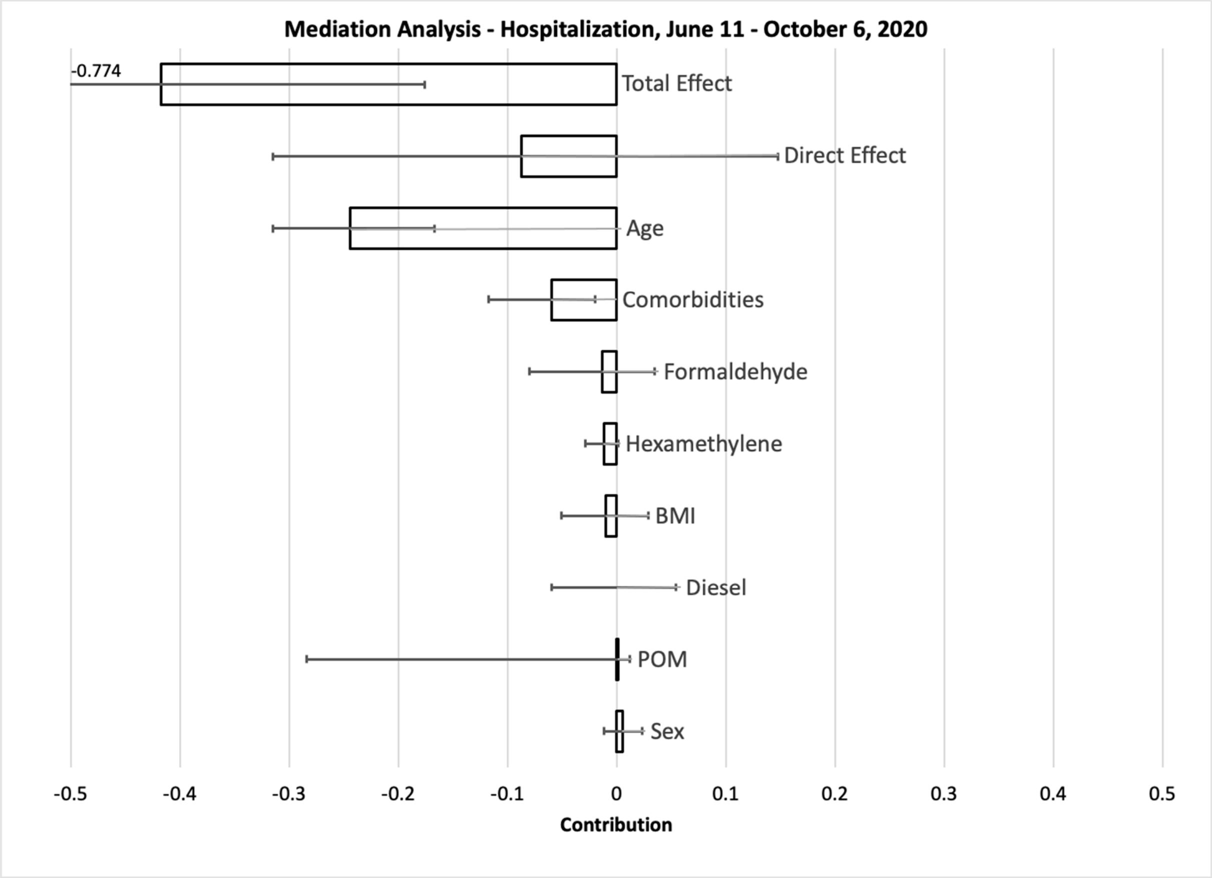

Figure S4: Mediation analysis results for hospitalizations, June 11 – October 6, 2020. Whiskers indicate the 95% confidence interval around the mediation effect, with each tested mediator shown by a column. Statistically significant effects are bolded. Positive total effect suggests a larger effect in Black patients compared with White patients, and a negative total effect suggests a larger effect in White patients compared with Black patients.

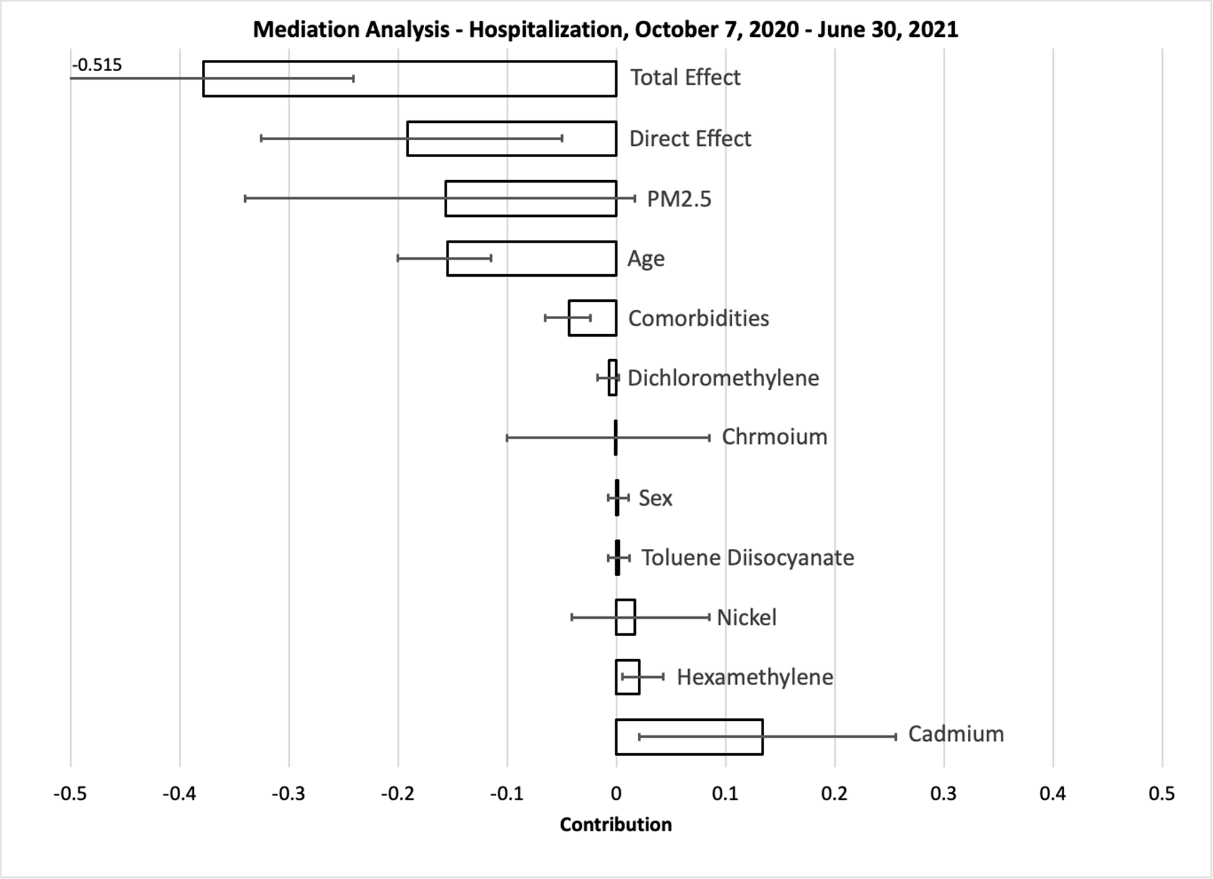

Figure S5: Mediation analysis results for hospitalizations, October 7, 2020 – June 30, 2021. Whiskers indicate the 95% confidence interval around the mediation effect, with each tested mediator shown by a column. Statistically significant effects are bolded. Positive total effect suggests a larger effect in Black patients compared with White patients, and a negative total effect suggests a larger effect in White patients compared with Black patients.

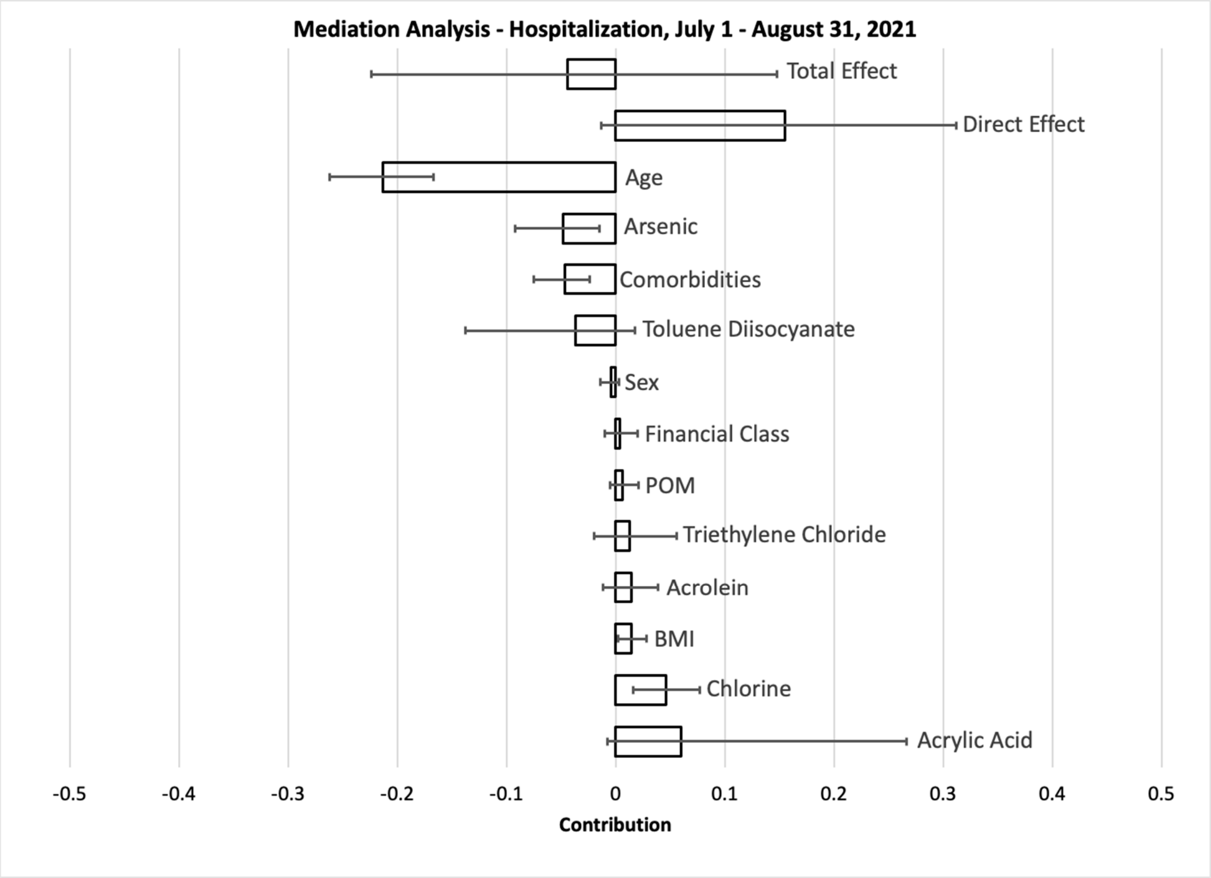

Figure S6: Mediation analysis results for hospitalizations, July 1 – August 31, 2021. Whiskers indicate the 95% confidence interval around the mediation effect, with each tested mediator shown by a column. Statistically significant effects are bolded. Positive total effect suggests a larger effect in Black patients compared with White patients, and a negative total effect suggests a larger effect in White patients compared with Black patients.

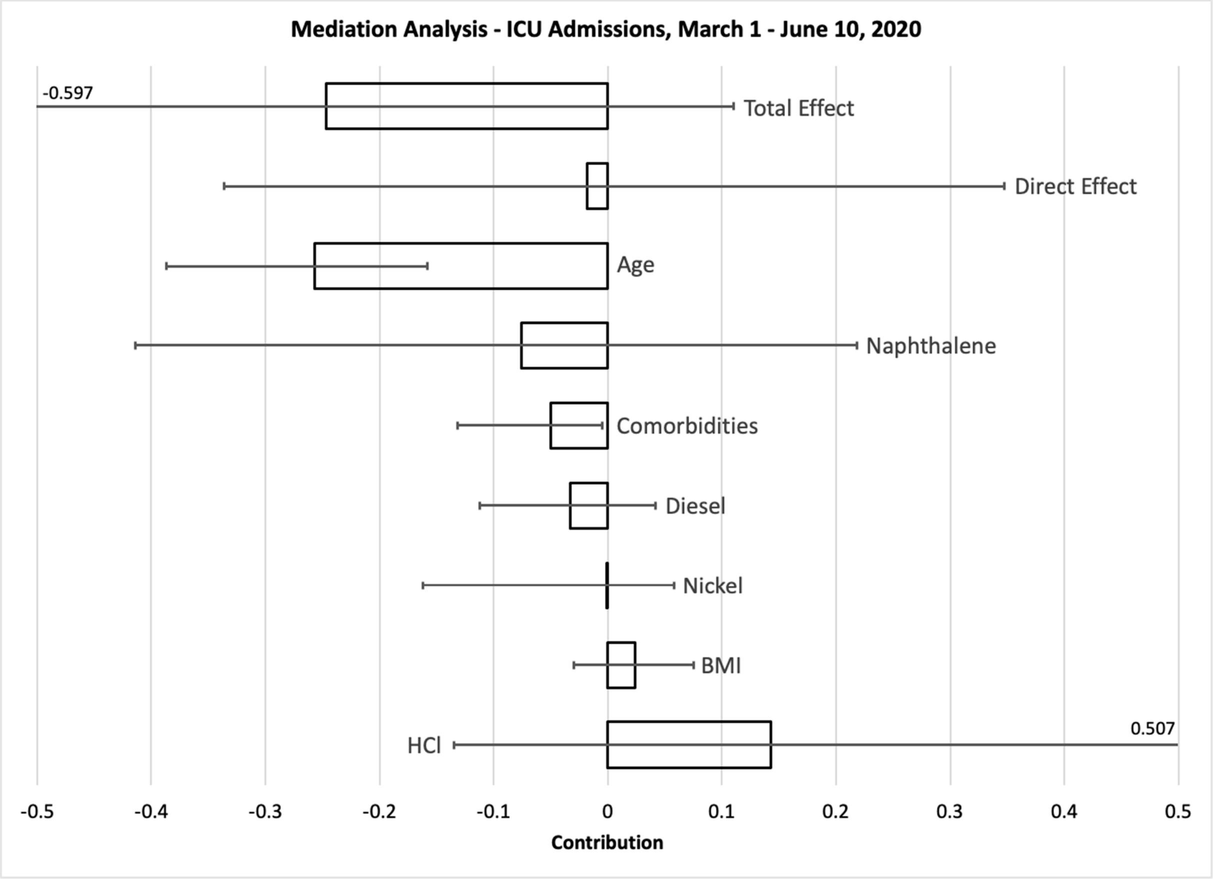

Figure S7: Mediation analysis results for ICU admissions, March 1 – June 10, 2020. Whiskers indicate the 95% confidence interval around the mediation effect, with each tested mediator shown by a column. Where the lower confidence interval goes beyond the data range shown on the page, the lower bound is provided numerically on the graph. Statistically significant effects are bolded. Positive total effect suggests a larger effect in Black patients compared with White patients, and a negative total effect suggests a larger effect in White patients compared with Black patients.

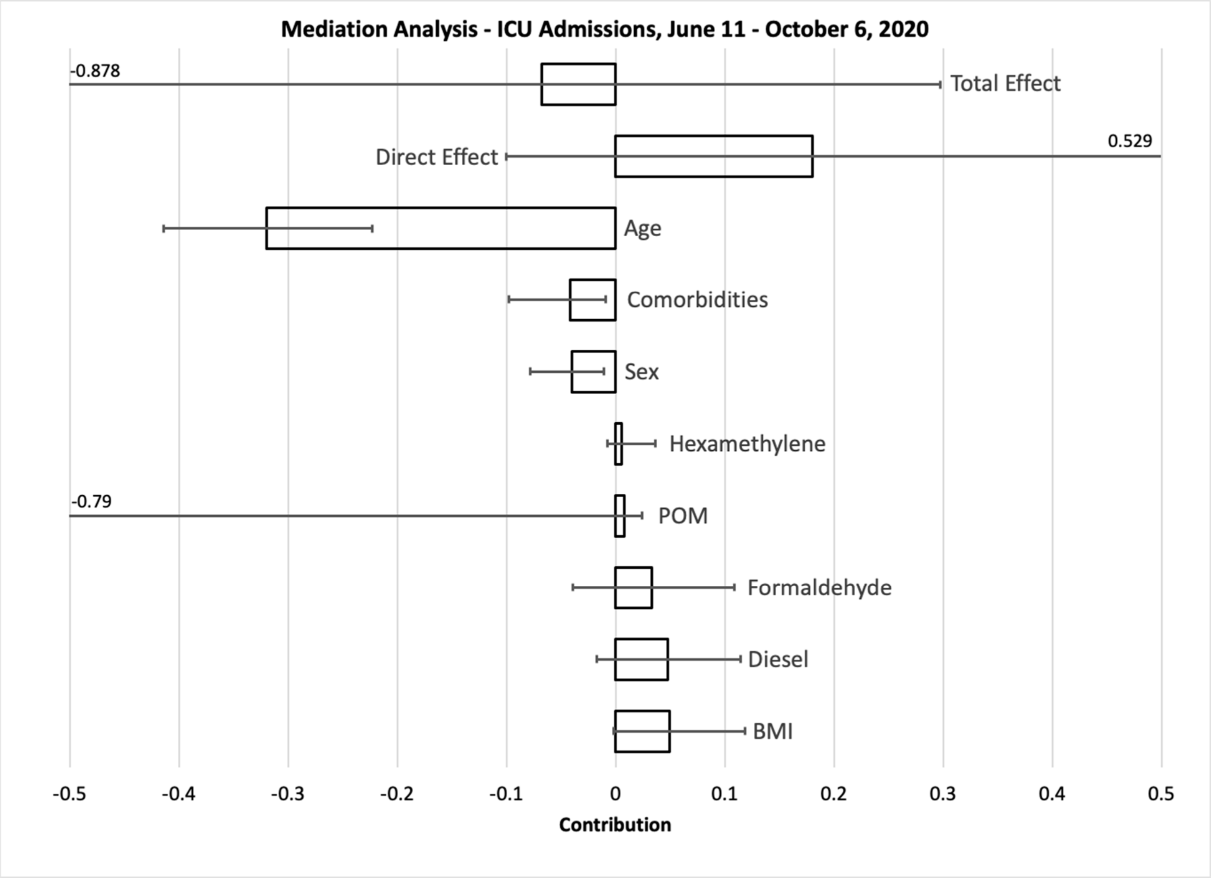

Figure S8: Mediation analysis results for ICU admissions, June 11 – October 6, 2020. Whiskers indicate the 95% confidence interval around the mediation effect, with each tested mediator shown by a column. Where the lower confidence interval goes beyond the data range shown on the page, the lower bound is provided numerically on the graph. Statistically significant effects are bolded. Positive total effect suggests a larger effect in Black patients compared with White patients, and a negative total effect suggests a larger effect in White patients compared with Black patients.

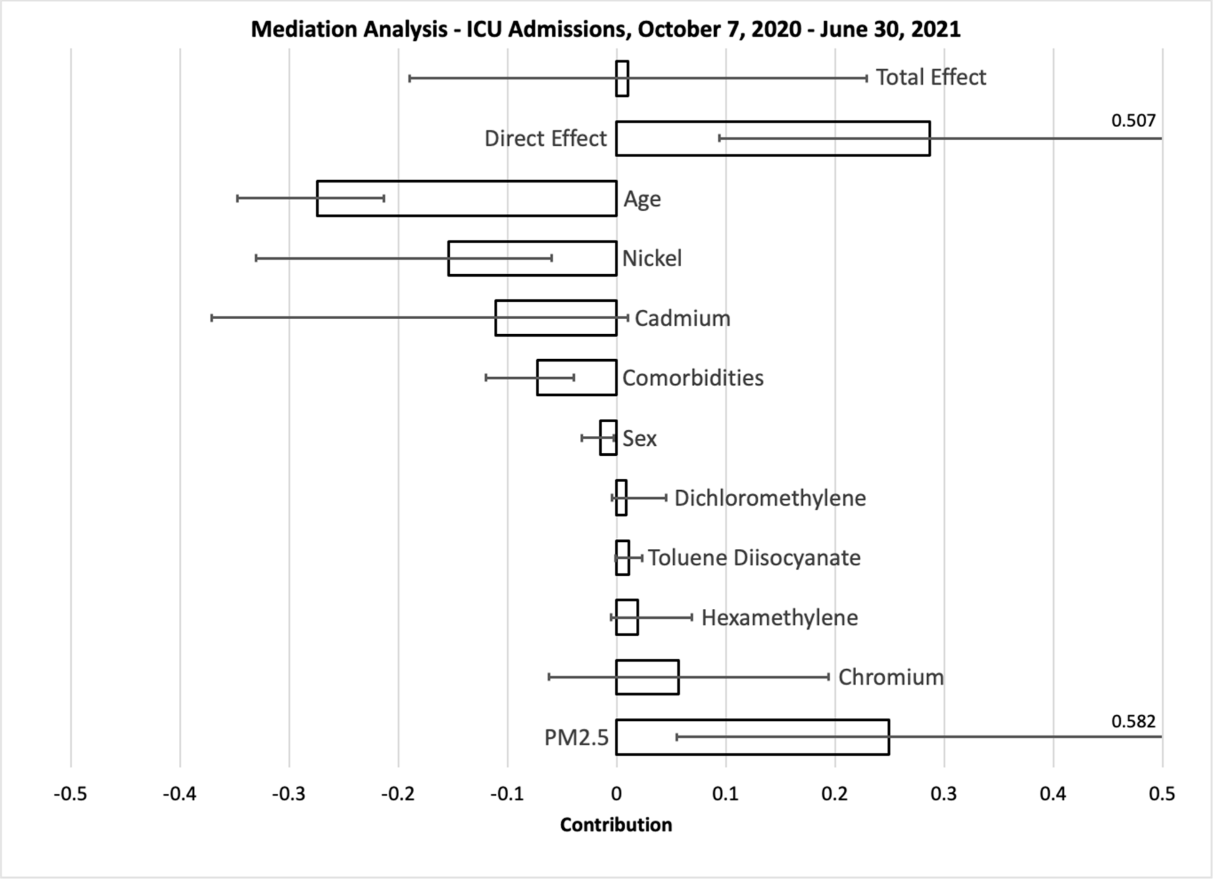

Figure S9: Mediation analysis results for ICU admissions, October 7, 2020 – June 30, 2021. Whiskers indicate the 95% confidence interval around the mediation effect, with each tested mediator shown by a column. Where the lower confidence interval goes beyond the data range shown on the page, the lower bound is provided numerically on the graph. Statistically significant effects are bolded. Positive total effect suggests a larger effect in Black patients compared with White patients, and a negative total effect suggests a larger effect in White patients compared with Black patients.

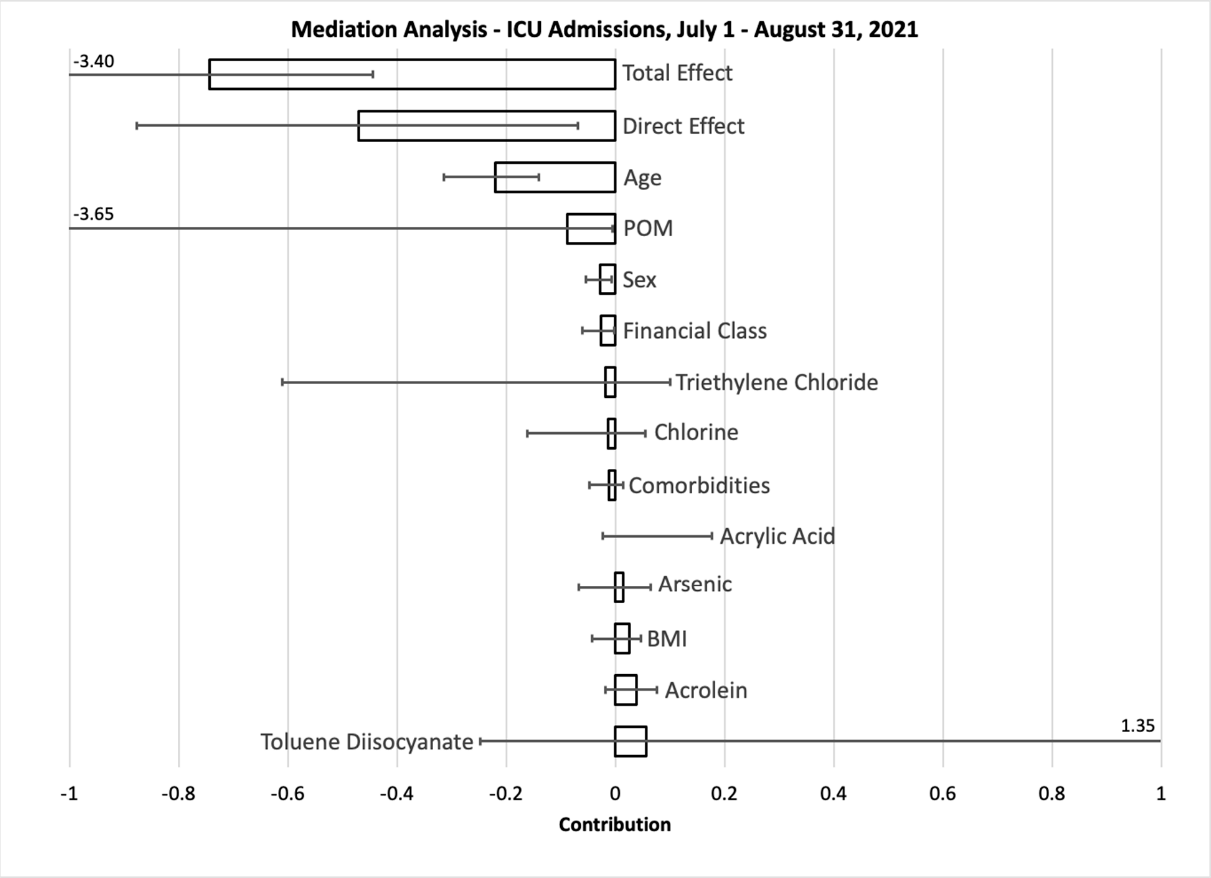

Figure S10: Mediation analysis results for ICU admissions, July 1 – August 31, 2021. Whiskers indicate the 95% confidence interval around the mediation effect, with each tested mediator shown by a column. Where the lower confidence interval goes beyond the data range shown on the page, the lower bound is provided numerically on the graph. Statistically significant effects are bolded. Positive total effect suggests a larger effect in Black patients compared with White patients, and a negative total effect suggests a larger effect in White patients compared with Black patients.

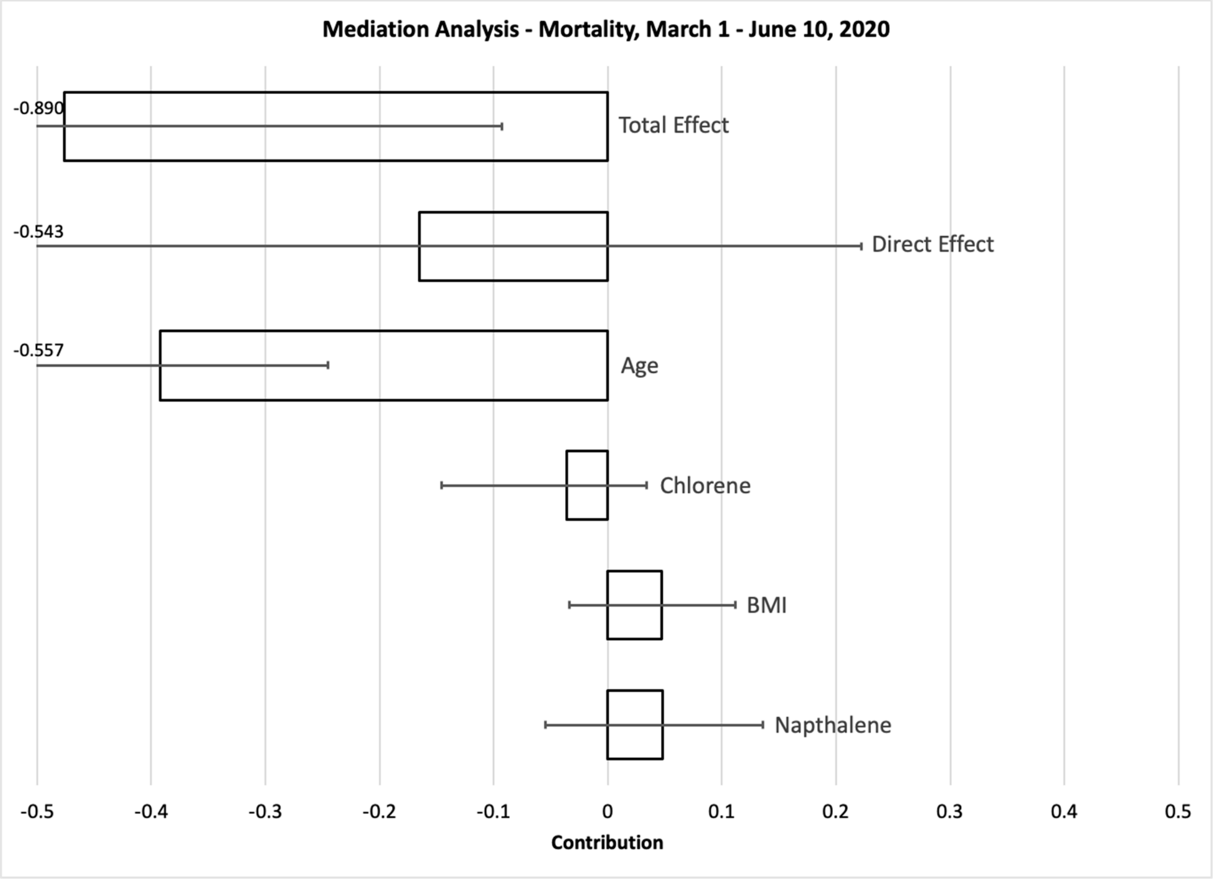

Figure S11: Mediation analysis results for mortality, March 1 – June 10, 2020. Whiskers indicate the 95% confidence interval around the mediation effect, with each tested mediator shown by a column. Statistically significant effects are bolded. Positive total effect suggests a larger effect in Black patients compared with White patients, and a negative total effect suggests a larger effect in White patients compared with Black patients.

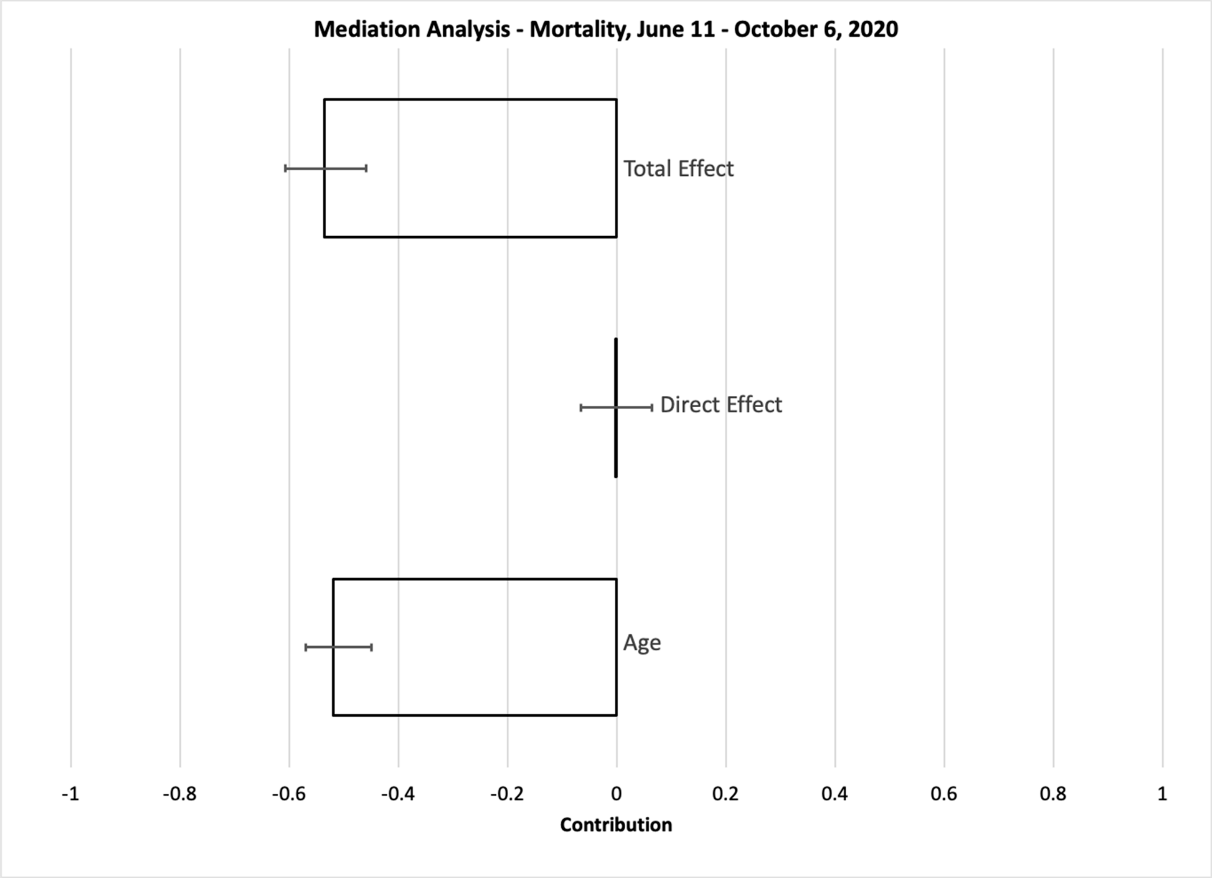

Figure S12: Mediation analysis results for mortality, June 11 – October 6, 2020. Whiskers indicate the 95% confidence interval around the mediation effect, with each tested mediator shown by a column. Statistically significant effects are bolded. Positive total effect suggests a larger effect in Black patients compared with White patients, and a negative total effect suggests a larger effect in White patients compared with Black patients.

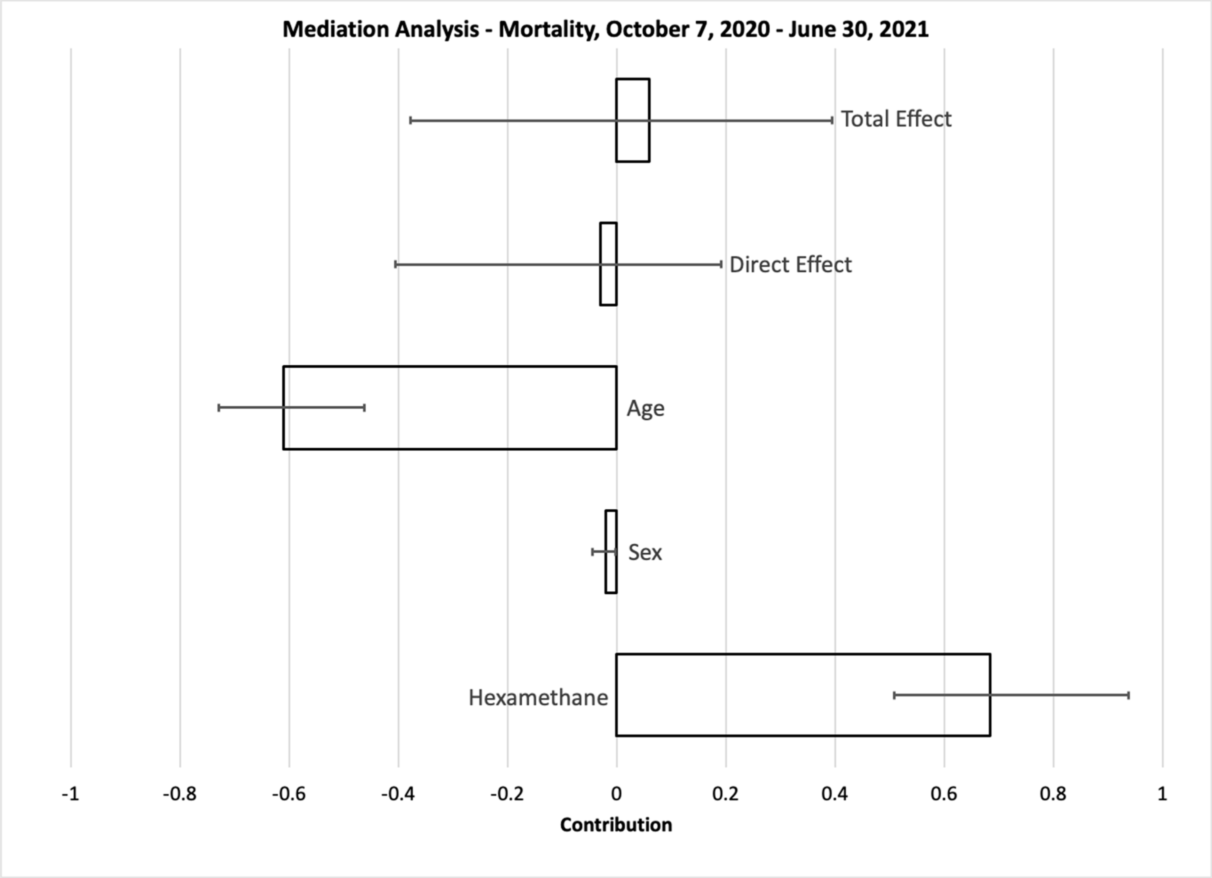

Figure S13: Mediation analysis results for mortality, October 7, 2020 – June 30, 2021. Whiskers indicate the 95% confidence interval around the mediation effect, with each tested mediator shown by a column. Statistically significant effects are bolded. Positive total effect suggests a larger effect in Black patients compared with White patients, and a negative total effect suggests a larger effect in White patients compared with Black patients.

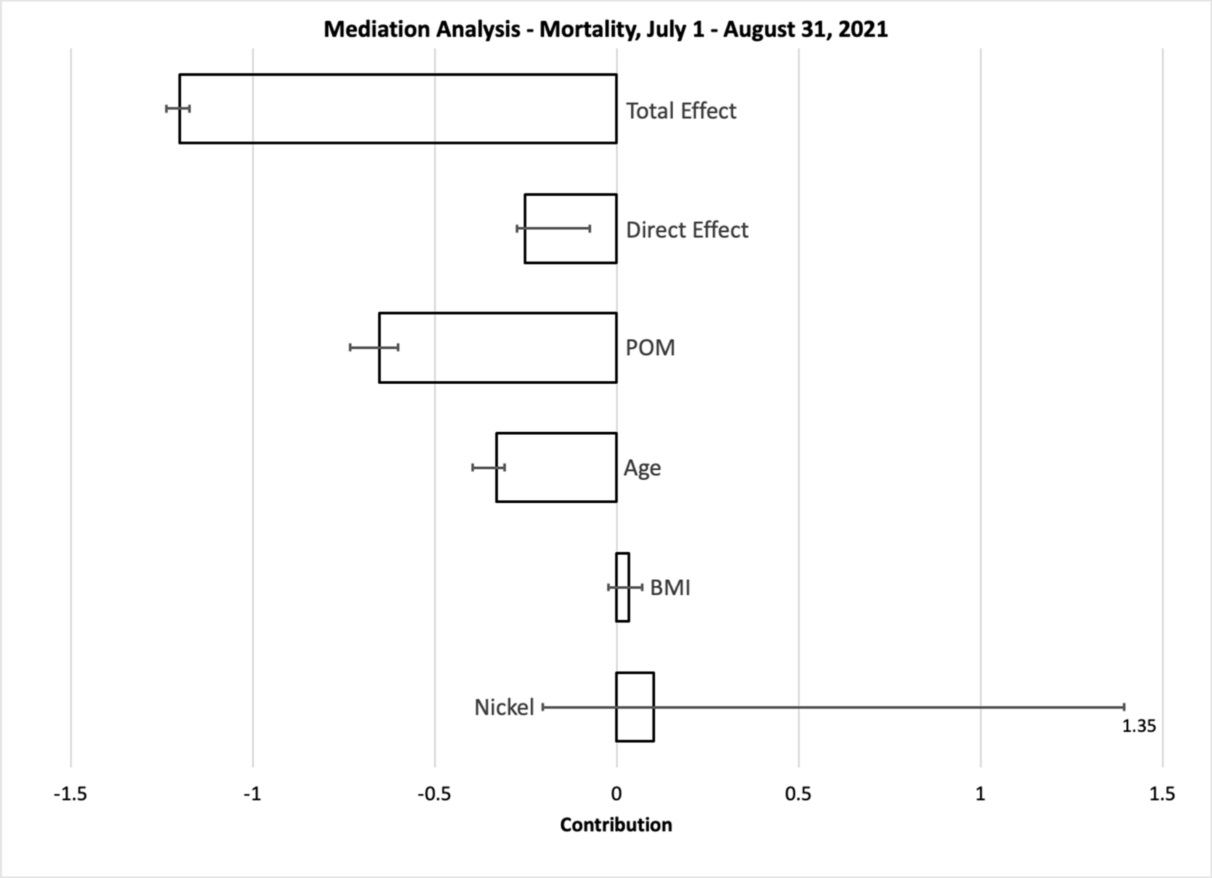

Figure S14: Mediation analysis results for mortality, July 1 – August 31, 2021. Whiskers indicate the 95% confidence interval around the mediation effect, with each tested mediator shown by a column. Statistically significant effects are bolded. Positive total effect suggests a larger effect in Black patients compared with White patients, and a negative total effect suggests a larger effect in White patients compared with Black patients.
